# Measurement of the Interaction Between Recombinant I-domain from Integrin alpha 2 beta 1 and a Triple Helical Collagen Peptide with the GFOGER Binding Motif Using Molecular Force Spectroscopy

**DOI:** 10.3390/ijms14022832

**Published:** 2013-01-29

**Authors:** Simon J. Attwood, Anna M. C. Simpson, SamirW. Hamaia, Dominique Bihan, Debdulal Roy, RichardW. Farndale, Mark E. Welland

**Affiliations:** 1 Nanoscience Centre, Department of Engineering, Cambridge University, Cambridge, CB3 0FF, UK; E-Mail: simonjamesattwood@gmail.com; 2 Department of Biochemistry, Cambridge University, Cambridge, CB2 1QW, UK; E-Mails: anna.simpson@cantab.net (A.M.C.S.); swh23@cam.ac.uk (S.W.H.); db422@mole.bio.cam.ac.uk (D.B.); 3 National Physical Laboratory, Teddington, TW11 0LW, UK; E-Mail: debdulal.roy@npl.co.uk

**Keywords:** integrin, alpha 2 beta 1, collagen, atomic force microscopy, single molecule force spectroscopy

## Abstract

The role of the collagen-platelet interaction is of crucial importance to the haemostatic response during both injury and pathogenesis of the blood vessel wall. Of particular interest is the high affinity interaction of the platelet transmembrane receptor, alpha 2 beta 1, responsible for firm attachment of platelets to collagen at and around injury sites. We employ single molecule force spectroscopy (SMFS) using the atomic force microscope (AFM) to study the interaction of the I-domain from integrin alpha 2 beta 1 with a synthetic collagen related triple-helical peptide containing the high-affinity integrin-binding GFOGER motif, and a control peptide lacking this sequence, referred to as GPP. By utilising synthetic peptides in this manner we are able to study at the molecular level subtleties that would otherwise be lost when considering cell-to-collagen matrix interactions using ensemble techniques. We demonstrate for the first time the complexity of this interaction as illustrated by the complex multi-peaked force spectra and confirm specificity using control blocking experiments. In addition we observe specific interaction of the GPP peptide sequence with the I-domain. We propose a model to explain these observations.

## 1. Introduction

The interaction of collagen with platelets plays a pivotal role in the haemostatic response [1,2]. During disease or injury endothelial cells that normally line blood vessel walls may become damaged, exposing the underlying collagens to the blood supply and permitting them to interact with the cellular components therein. Initially, the platelet receptor complex Glycoprotein Ib/V/IX (GpIb) binds indirectly to the exposed collagen surface mediated via immobilised von Willebrand Factor (VWF) [3]. This relatively weak interaction causes previously fast flowing platelets to roll in the direction of shear flow across the VWF-collagen surface as GpIb-VWF bonds are continuously formed and ruptured in front of and behind the platelet respectively. Rolling of the platelet ceases after firm attachment is achieved via integrin α_2_β_1_-collagen interaction. This firm attachment is required for the collagen to interact with the low affinity GpVI receptor which is mainly responsible for the collagen induced platelet activation. After platelet activation plasma fibrinogen and VWF may then bind to the α_IIb_β_3_ integrin receptors of adjacent platelets, securing the platelet plug in place and preventing more blood loss.

If any component of this sequential process is either absent or defective, there may be catastrophic consequences. For example, Bernard–Soulier syndrome is a bleeding disorder caused by a deficiency of GpIb receptors on platelets, and von Willebrand disease, characterised by a tendency to bleed after minor trauma, is caused by a lack of functional VWF. Other problems arise when arteries develop atherosclerotic plaques, which cause endothelial cell surfaces to become activated, initiating platelet attachment, platelet activation and thrombus formation. In some cases the plaque ruptures, revealing collagens which recruit and activate nearby platelets as described above. If thrombus formation occurs in vital blood vessels, such as the coronary artery or cerebral vasculature, then a heart attack or stroke may occur. It should be clear therefore that a thorough understanding of all these processes is a prerequisite for any potential treatment or prevention strategy.

For the purposes of this study we focus attention on the high affinity interaction of collagen [4,5] with the platelet receptor integrin α_2_β_1_, and in particular the α_2_ I-domain which has been shown to recapitulate the adhesion properties of the entire receptor [6]. The integrins are heterodimeric transmembrane receptors with the subunits being held together by non-covalent interactions [7]. Integrins in general have been studied extensively using conventional ensemble techniques [8]. However, only limited studies of integrin-collagen interactions at the molecular level have thus far been conducted.

The technique of Single Molecule Force Spectroscopy (SMFS) [9] has been used to measure the interaction between single integrin molecules and their respective ligands using the Atomic Force Microscope (AFM) [10]. These include integrin α_L_β_2_ with its cognate ligand intercellular adhesion molecule-1 [11], integrin α_5_β_1_ with fibronectin [12–15] and integrin α_IIb_β_3_ with fibrinogen [16]. These experiments either employ single-cell force spectroscopy (SCFS), for which single cells expressing integrin receptors are attached to AFM cantilever tips, or else recombinant protein forms are immobilised directly onto surfaces. Recently Taubenberger *et al*. [17] utilised SCFS to study the adhesion between cells expressing α_2_β_1_ and a collagen type I matrix. They first demonstrated that the mean maximal detachment force for successive approach-retract cycles was significantly greater for the cells expressing the integrins as compared with the wild type cells, which did not. Then, by pressing the cells onto the collagen substrate with a decreased force, they were able to reduce the contact area to the extent that they predict will permit measurement of the rupture of individual integrin receptor-substrate bonds. They observed unimodal force spectra, indicated by the close fit to Gaussian distributions.

Several different regions of the collagen have been identified [18] as interacting with different affinities towards α_2_β_1_. Previous studies utilised synthetic triple helical peptide sequences containing specific recognition motifs and flanked on either side by [GPP]*_n_* host sequences which impart triple helical structure to the specific, guest, sequence. These studies have identified the GFOGER peptide as being a high-affinity ligand for α_2_β_1_ [19], as well as other sequences such as GLOGER, GMOGER and GAOGER of varying affinity [18,20,21]. This may suggest therefore that the unimodal force spectra observed actually reflects an average of several different, but presumably similar interactions of collagen with the receptor.

In the current work we utilise a recombinant form of the I-domain from α_2_β_1_ presented in its “locked- open” or high affinity state (LOI) [21], in conjunction with a synthetic triple helical peptide containing the GFOGER binding motif. The I-domain was attached to template stripped gold (TSG) substrates, and the peptide sequence to a gold coated cantilever via a flexible poly(ethylene) glycol (PEG) heterobifunctional crosslinker using a protocol developed recently in our laboratory to minimise non-specific adhesion [22]. We were able to tether the I-domain via a cysteine residue at its base and therefore control orientation. We probed the interaction of the I-domain with both the GFOGER peptide, and a GPP peptide containing no guest residues at the single molecule level.

## 2. Results and Discussion

**Figure 1:**
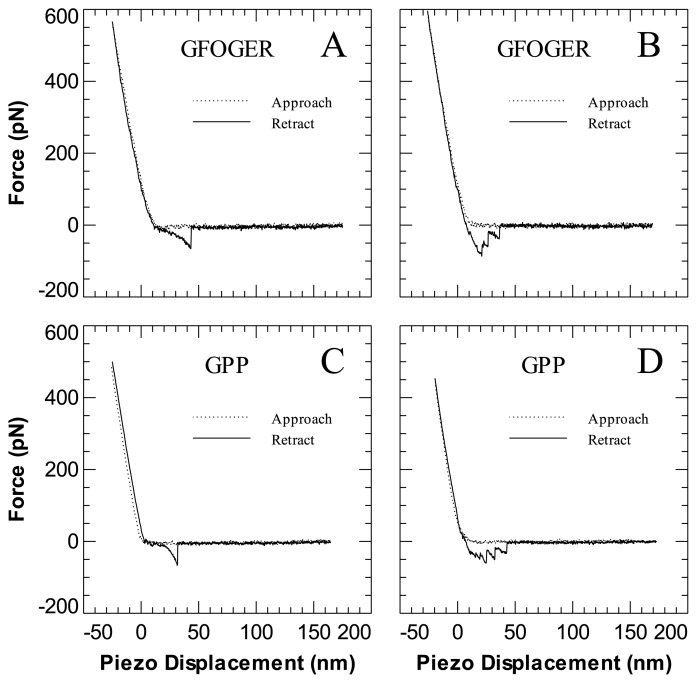
Representative force-displacement curves illustrating the interaction of the I-domain with either GFOGER or GPP. (**A**) Single or multiple simultaneous unbinding of GFOGER; (**B**) Sequential unbinding of GFOGER; (**C**) Single or multiple simultaneous unbinding of GPP; (**D**) Sequential unbinding of GPP. All types of curve may be observed within an experiment.

A variety of force-displacement curves were observed for the interaction of the I-domain with either GFOGER or GPP as shown in Figure 1. Figure 1A shows an example of a single or multiple simultaneous unbinding event, characterised by the delayed non-linear extension profile immediately preceding rupture. The non-linear form is widely considered to be associated with the extension of the flexible PEG linkers [23,24] and is a useful indicator of the specificity of the unbinding. In Figure 1B an example of sequential unbinding between the I-domain and GFOGER is shown as indicated by the canonical cascade of rupture events. Most interesting however is that there appears to be some specific interaction between the I-domain and GPP peptide as illustrated again by the characteristic non-linear retraction profile shown in Figure 1C. Sequential unbinding is also observed for this pair as shown in Figure 1D.

**Figure 2:**
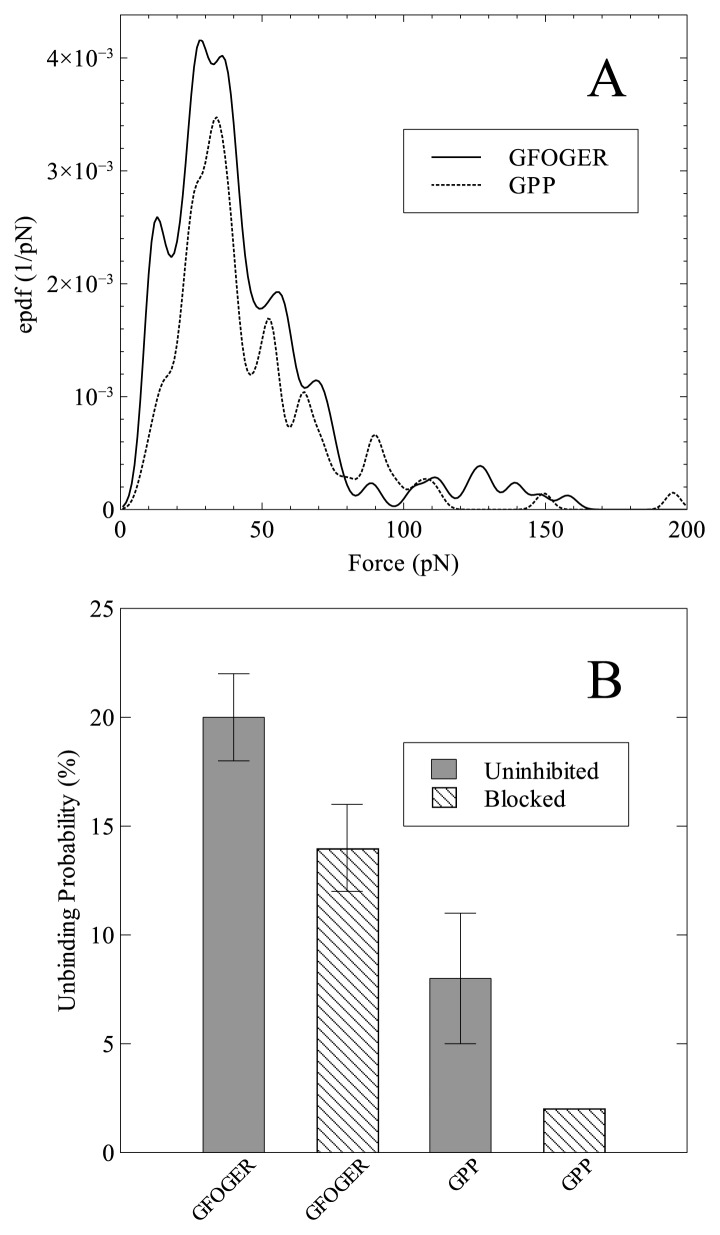
(**A**) Representative force spectra for the interaction of I-domain with GFOGER (solid line) and GPP (dashed line). The spectra correspond to 190 (GFOGER) and 125 (GPP) individual F-D curves respectively; (**B**) Specificity tests illustrating the unbinding probability observed for both uninhibited (solid grey) interactions and after inhibition with free I-domain (dashed shading). Both the GFOGER and GPP experiments were repeated four times using fresh cantilevers and the reported values represent means of these experiments.

Figure 2A shows two representative force spectra for the interaction of the I-domain with GFOGER (solid line) and GPP (dashed line). Both spectra are comprised of a single large broad peak spanning 0–100 pN with a series of smaller sub-peaks superimposed. The particular form of the spectra contrasts with other interactions reported in the literature, such as the model biotin-avidin pair, where predominantly only one or two large peaks are observed over similar force ranges [22,25–29], including our measurements using the same instrument used here. In order to test the specificity of the two interactions blocking tests were conducted in which free I-domain was flowed into the AFM fluid cell in order to block the GFOGER and GPP peptides. For each data set four new cantilevers were used and the values given represent means. When blocking the GFOGER peptide a small (one third) reduction in unbinding probability from 20% to 14% was observed. The unbinding probability for the uninhibited interaction of GPP with the I-domain was found to be 8%. Upon blocking, this decreased by three-quarters, from 8% to 2%. The decrease in unbinding probability when comparing the uninhibited interaction of GFOGER with I-domain to that of the blocked interaction of GPP was 90%. This strongly indicates that specific interactions were being measured since the GFOGER peptide contains GPP sequences as well, and therefore the large reduction can only be attributed to the absence of the specific GFOGER sequence. Two things however require further explanation; firstly that specific interactions were measured at all for the GPP ligand and secondly that the reduction in unbinding probability was only 30% when blocking the GFOGER ligand.

Previously it had been shown [18] when comparing various peptide sequences using enzyme-linked immunosorbent assay (ELISA) that the GFOGER peptide interacts most strongly with the I-domain. In contrast the GPP peptide, lacking any specific guest sequences, showed so little adhesion that it was often considered as a negative control. However, the absorbance and therefore adhesion observed for the GPP interaction in that work was small but not zero, and it was also greater than the interaction of I-domain with BSA (bovine serum albumin), another negative control for which there is no basis for specific interaction. Furthermore, a weak interaction between GPP and GPVI has also recently been proposed [30].

**Figure 3:**
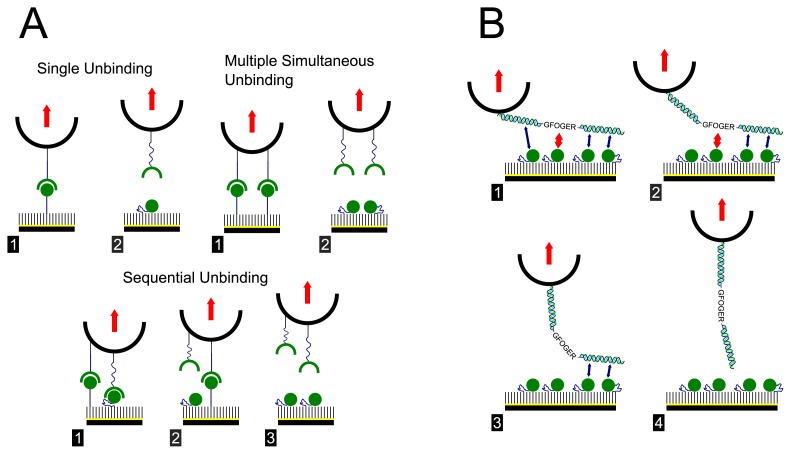
Schematics illustrating possible interactions of tip bound ligands and substrate bound receptors as in typical SMFS experiments. (**A**) Possible interactions associated with a simple ligand-receptor pair such as biotin-avidin; (**B**) Illustration of possible interactions with the triple helical GFOGER peptide and the I-domain. The ligand is thought to interact strongly with the GFOGER hexapeptide sequence, but may also interact with the flanking GPP residues.

A possible explanation for both the observed complexity in the force spectra and the relatively low reduction in unbinding probability when blocking the GFOGER peptide may be found when considering the molecular origins of the interaction force. For a simple model ligand-receptor pair such as biotin-avidin the complementary binding site is very specific and localised. In the case of an optimal experiment, only unbinding with an individual pair should be expected, however due to the difficulties associated with these type of experiments, both multiple simultaneous and sequential unbinding events may occur (Figure 3A). Such events help to explain the occurrence of a small number of additional peaks as observed previously. The current interaction however is inherently expected to be more complex, since the previously reported adhesion assay data indicates that both the GFOGER sequence and the flanking GPP residues may interact with the I-domain [18]. Although the GPP interaction is thought to be weak, at the molecular level it is possible that multiple bonds may form with the long, triple helical strands, at various points along the ligand. Sequential unbinding of the GFOGER peptide, with these concepts in mind, is illustrated schematically in Figure 3B. An array of different configurations may be proposed including combinations of both multiple simultaneous and sequential unbinding that therefore give rise to slightly different detachment forces leading to the multi-peaked force spectra observed. Such a situation would also mean that attempts to block the ligand would be quite difficult due to the number of available binding regions and because of steric hindrance.

**Figure 4:**
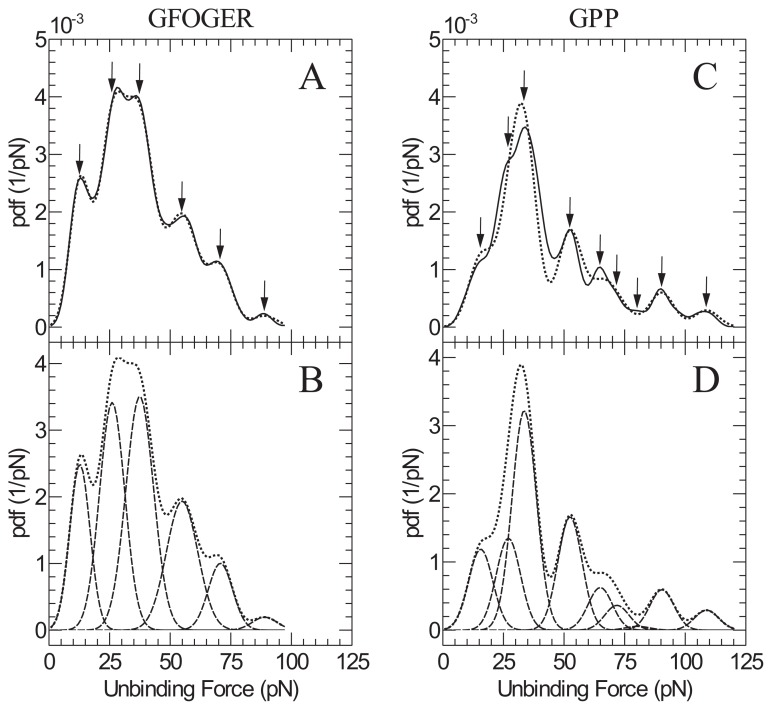
Peak analysis of probability distributions for both GFOGER (**A**,**B**) and GPP (**C**,**D**). Solid lines represent raw data whereas the dotted line is the best fit solution of a sum of several individual Gaussian distributions. Dashed lines in B and D correspond to the individual Gaussian components. Identified peaks are highlighted by arrows.

In order to further analyse the two data sets, a multi-component analysis was performed as shown in Figure 4. A model comprising of a sum of six and nine Gaussian components was constructed for the GFOGER and GPP data sets respectively. As can be seen, both data sets are reasonably well fit to the model and therefore estimates of the component peak heights and standard deviations were obtained as summarised in Table 1. Identifying any single peak that represents specific interaction of the hexapeptide sequence alone is very difficult. It appears in fact that many of the small sub-peaks occur at very similar forces for both the GFOGER and the GPP data. The role of GPP sequences in the interaction with I-domain therefore at this loading rate plays a much more significant role than might have been expected.

**Figure 5:**
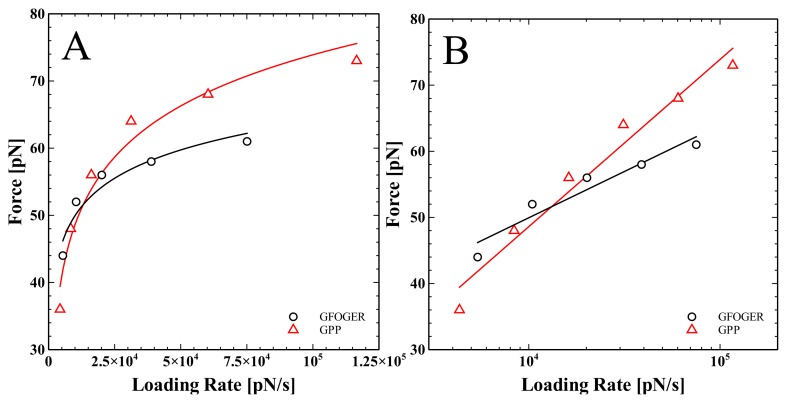
Force spectroscopy experiments were repeated over a range of different loading rates for the interaction of both GFOGER and GPP with the I-domain. For each loading rate the maximum force of the most dominant peak in the force spectra was chosen and plotted against the loading rate. Data for GFOGER/I-domain (black) and GPP/I-domain (red) interactions are plotted on both a linear x-axis (A) and a logarithmic x-axis (B). Using the Bell–Evans model we find for the GFOGER interaction the kinetic off rate, *k*_off_ = 0.44 s^−1^ and ground state to transition state distance, *x_β_* = 0.7nm. For the GPP interaction we find *k*_off_ = 11 s^−1^ and *x_β_* = 0.37nm.

Another point that should be addressed is the similarity in unbinding force found between the different peptides in the current work as compared with the large difference in adhesion observed using the ELISA assay. The key point is that these measurements were conducted at a single loading rate and therefore represent a single point on a dynamic spectrum of forces [25]. Although a similar magnitude of unbinding force was observed at this specific loading rate, it is likely that the unbinding forces will diverge across the loading rate spectrum. We therefore decided to conduct dynamic force spectroscopy measurements by repeating the experiments over a range of different loading rates as shown in Figure 5.

Such experiments may be modelled by the Bell–Evans model [31–33]:

(1) $$F^{*}(r)=\frac{k_{\text{B}}T}{x_{\beta }}\text{ln}\frac{rx_{\beta }}{k_{\text{off}}k_{\text{B}}T}$$

This model assumes a single energy barrier and demonstrates that the most probable rupture force, F* (*r*), is logarithmically dependent on the loading rate, *r*. By fitting this equation to the experimental data we can find the kinetic off rate, *k*_off_, which is the inverse of the ligand-receptor bond lifetime, *k_off_* (0) = 1/τ (0) at zero applied force, and the distance between the ground state and transition state, *x_β_*. For both the GFOGER/I-domain interaction and the GPP/I-domain interaction, we observe a linear dependence of the rupture force on the logarithm of the loading rate. Such logarithmic dependence is another indication that we are measuring specific ligand-receptor interactions. For GFOGER we find *k*_off_ = 0.44 s^−1^, *x_β_* = 0.7nm and for GPP we find *k*_off_ = 11 s^−1^ , *x_β_* = 0.37nm. This suggests that the bond lifetime for GFOGER is much longer than for GPP at zero applied force. However, when GPP is loaded quickly (above the cross-over point *r* = 1.6 *×* 10^4^ pN s^−1^ ), it resists much more than GFOGER.

It seems likely that the identity between the force curves may represent the unbinding of GPP triplets, which are common to both peptides. The underlying molecular interaction may reflect the ability of the GPP triple helix to fit the same binding groove on the I-domain as the specific peptide, GFOGER. The difference between the two spectra may reflect the unbinding of the specific GFOGER hexapeptide itself. A crystal structure of the GFOGER/I-domain complex [6] revealed a binding groove long enough to accommodate little more than the two triplets, GFO and GER. Interactions were mainly between the negatively-charged glutamate residue, the positively-charged arginines and the hydrophobic phenylalanines, but in addition, several interactions occurred with the polypeptide main chain within these two triplets that would also be possible if GPP polymers were docked onto the I-domain’s collagen binding site. Thus it seems plausible that the common peaks in the spectra may reflect unbinding of such interactions, and be accounted for in the case of unbinding of GFOGER by mis-registration between the peptide and the I-domain such that the flanking GPP triplets occupied the binding groove rather than the specific GFOGER sequence.

## 3. Experimental Section

### 3.1. Materials and Instrumentation

HS-(CH_2_)_11_-(O-CH_2_-CH_2_)_6_-NH_2_ (Alkyl-PEG-Amine, APA) was purchased from Prochimia (Gdansk, Poland). HS-(CH_2_)_10_-CO-NH-(CH_2_)_2_(O-CH_2_-CH_2_)_7_-OH (Alkyl-PEG-Hydroxyl, APH) and the crosslinker, maleimidopropionyl-PEG-NHS were purchased from Polypure (Oslo, Norway). Traut’s reagent (2-Iminothiolane hydrochloride), phosphate buffered saline (PBS: 10 mM phosphate, 138 mM NaCl, 2.7 mM KCl, pH 7.4), dimethyl sulfoxide and absolute ethanol were purchased from Sigma-Aldrich. Tris-buffered saline (TBS) was prepared with 50 mM Tris-HCl, 140 mM NaCl, pH = 7.4. Immulon 2B 96-well plates were purchased from Fisher and 12 well plates were purchased from Corning. Epotek 377 epoxy was purchased from Promatech (Gloucestershire, UK) or John P. Kummer (Marlborough, UK). Silicon *<*100*>*, 500 *μ*m thick test grade wafers were purchased from Compart Technology (Peterborough, UK), Gold wire (99.99%) was obtained from Advent Research Materials (Oxford, UK) and muscovite mica from Agar Scientific (Essex, UK). Gold coated cantilevers were purchased from Olympus (Biolevers BL-RC150VB). The GFOGER and GPP peptides were synthesised as described elsewhere [18]. The full sequences of these peptides are: GPC[GPP]_10_GPCG and GPC[GPP]_5_GFOGER[GPP]_5_GPCG. A high affinity form of the I-domain, locked open I-domain (LOI), was prepared as detailed previously [21] but was additionally cleaved of its GST tag to help reduce non-specific interactions (CLOI).

### 3.2. Preparation of Template-Stripped Gold (TSG) Surfaces

Template-stripped gold substrates were prepared using a similar process to that described by Wagner *et al*. [34,35]. Briefly, freshly cleaved muscovite mica sheets were placed in a BOC Edwards Auto 306 evaporator and heated to 300 °C in situ from the rear through the sample holder, for approximately 6–12 h at a pressure less than 10^−6^ mBar. Whilst maintaining 300 °C, ~ 200nm gold films were deposited. Samples were annealed in the evaporator again at 300 °C typically for a further 6 h and allowed to cool naturally before removing from the chamber. Silicon stubs were then glued onto the gold coated side of the mica using Epo-tek 377 two-part epoxy. The samples were cured for approximately 48 h at 70 °C. Immediately prior to use the mica was stripped from the TSG samples by gently bending the excess gold-mica layer normal to the silicon stub. Typically this yielded clean flat substrates (rms roughness ~ 0.5nm).

### 3.3. Monolayer Preparation

Covalent attachment of the I-domain and the triple helical peptides (GFOGER, GPP) was achieved using a similar protocol described recently [22]. Both the I-domain and the peptides contain cysteine residues with free sulfhdryl groups and as such the use of Traut’s reagent was not necessary in this case.

**Substrate:** Freshly stripped TSG samples were functionalised by incubation in 200 *μ*L of 1mM APA in ethanol for 1 h. Samples were then washed in ethanol and dried under a stream of nitrogen. Samples were then incubated in 200 *μ*L of 2mM crosslinker in PBS (diluted from 100mM stock in DMSO) for 1 h followed by washing using PBS to remove any unreacted crosslinker. Finally the samples were allowed to react with 200 *μ*L of 1 *μ*M of CLOI in TBS for 1 h. Substrates were then washed using TBS and care taken to ensure that the proteins were not dehydrated.

**Cantilevers:** A 100 *μ*L solution containing 0.1mM APA and 0.9mM APH (10% APA/APH ratio) was added to one well of a 96-well plate, before carefully submerging a gold coated cantilever. After incubating the lever for 1 h it was carefully washed in ethanol and allowed to dry evaporatively. Next, 100 *μ*L of 2mM crosslinker in PBS (diluted from 100mM stock in DMSO) was added to a fresh well and the lever again carefully submerged. After a further hour the tip was thoroughly washed in PBS and any excess buffer wicked away from the chip using cleanroom wipes. Finally, 100 *μ*L of either 4.5 *μ*M GFOGER or GPP in TBS was added to a new well and the cantilever again submersed. After 1 h the cantilever was then washed with copious amounts of TBS to remove any unbound molecules.

### 3.4. Atomic Force Microscopy: Single Molecule Force Spectroscopy

A PicoPlus AFM with a PicoSPM II controller from Molecular Imaging was used for all force spectroscopy work. Cantilever spring constants were estimated using the Sader Method [36] typically yielding values about 0.03Nm^−1^, consistent with nominal values quoted by the manufacturer. Calibration of gold coated cantilevers was always performed before any chemical modification.

All force spectroscopy measurements were conducted in a fluid cell in PBS. Modified tips were repeatedly approached towards and retracted away from the modified substrate in a cyclic manner whilst simultaneously monitoring the deflection signal using a four quadrant photodiode. Amplitudes were typically 200nm with a frequency of 1 Hz. Approach-retract cycles were performed 1000 times with each tip for each experiment across five different areas of the substrate. Typically an uninhibited experiment would be conducted first, followed by a blocking experiment in which free avidin was introduced into the fluid cell. Force-distance cycles were analysed using purpose written software created using MATLAB version R2007a (MathWorks Inc., Natick, USA). Briefly, an algorithm first identifies any data points that deviate significantly from the rest of the data by comparing the gradient between successive points to the standard deviation of the gradient of all points. The user then chooses an event if it is preceded by a non-linear force-displacement retraction profile that is unimpeded by any other interaction. If no such event meets this criterion, the data set is discarded. Empirical Probability density functions [24,37] were then constructed from the distribution of unbinding forces. This representation is advantageous over simple histograms since each data point is weighted by their accuracy (essentially governed by the thermal noise of the cantilever fluctuations) and therefore yields better resolution. In order to determine the loading rate, we fitted the worm-like-chain (WLC) model to the retraction curve (force versus tip-sample displacement) as described elsewhere [24]. The gradient at the point immediately prior to rupture is then equal to the spring constant of the flexible linker, *k*_PEG_. Thus the effective spring constant was determined by *k*_eff_ = (*k*_PEG_^−1^ + *k_c_*^−1^) ^−1^, where *k_c_* is the cantilever spring constant and the loading rate found from *r* = *k*_eff_*v*, where *v* is the retraction speed.

## 4. Conclusions

We have, for the first time, measured the interaction between a recombinant form of the α_2_β_1_ I-domain and a triple helical collagen peptide with the GFOGER binding motif using the technique of single molecule force spectroscopy. As a comparison we also measured the interaction of the I-domain with a GPP peptide sequence that lacked the guest residues. We utilised a recently developed protocol for attaching both the I-domain and the peptides to substrate and tip respectively. Control experiments in which the peptides were blocked with free I-domain were also conducted. A decrease in unbinding probability when blocking from 20% to 14% for the GFOGER interaction and from 8% to 2% for the GPP interaction indicated that specific interactions were being measured. However, although the unbinding probability was reduced when comparing the uninhibited GFOGER and GPP, it was not completely eliminated. Indeed, force-displacement curves exhibiting the characteristic delayed, non-linear retraction profile for the GPP interaction were indicative of specific unbinding. We therefore proposed a model to describe these observations in which the GFOGER peptide may interact with the I-domain not only via the hexapeptide sequence, but also at any point along the host GPP residues. Such a model would explain the observation of a multitude of force-displacement curves exhibiting both single/multiple simultaneous unbinding events as well as sequential rupture profiles. Such an array of unbinding is manifested by the complex multitude of small sub-peaks superimposed on the broad force spectrum for both the GFOGER and GPP interactions. Such observations are compatible with previous adhesion assay measurements in which it was found that the adhesion of the I-domain was much greater with GFOGER than with the GPP sequence, since we are probing only a single point on the dynamic force spectrum.

We believe the application of the recently developed protocol for the tethering of both the recombinant form of the I-domain and the peptide sequences for use in single molecule force spectroscopy is a novel approach. The significance of the GPP residues in this particular interaction under these particular conditions has also not been reported previously.

## Figures and Tables

**Table 1:** Summary of peak forces for GFOGER and GPP.

	GFOGER	GPP
Peak	Peak force (pN)	Peak force ( pN)
1	13 *±* 2	16 *±* 2
2	26 *±* 4	27 *±* 4
3	37 *±* 6	34 *±* 5
4	55 *±* 8	52 *±* 8
5	71 *±* 11	65 *±* 10
6	89 *±* 13	72 *±* 10
7	-	80 *±* 12
8	-	90 *±* 14
9	-	109 *±* 16
